# Growth Restriction in the Offspring of Mothers With Polycystic Ovary Syndrome

**DOI:** 10.1001/jamanetworkopen.2024.30543

**Published:** 2024-08-27

**Authors:** Maren Sophie Aaserud Talmo, Ingvild Skogedal Fløysand, Guro Ørndal Nilsen, Tone S. Løvvik, Rønnaug Ødegård, Petur Benedikt Juliusson, Eszter Vanky, Melanie Rae Simpson

**Affiliations:** 1 Department of Clinical and Molecular Medicine, Faculty of Medicine and Health Sciences, Norwegian University of Science and Technology, Trondheim, Norway; 2 St Olavs Hospital, University Hospital of Trondheim, Trondheim, Norway; 3 Department of Obstetrics and Gynecology, St. Olavs Hospital, Trondheim University Hospital, Trondheim, Norway; 4 Centre for Obesity Research, St Olavs Hospital, Trondheim University Hospital, Trondheim, Norway; 5 Department of Health Registry Research and Development, National Institute of Public Health, Bergen, Norway; 6 Department of Clinical Science, University of Bergen, Bergen, Norway; 7 Department of Paediatric and Adolescent Medicine, Haukeland University Hospital, Bergen, Norway; 8 Department of Public Health and Nursing, Norwegian University of Science and Technology, Trondheim, Norway

## Abstract

**Importance:**

Polycystic ovary syndrome (PCOS) is a common endocrine disorder, characterized by subfertility, increased risk of metabolic diseases, and pregnancy complications. Previous studies diverge regarding the association between maternal PCOS and newborn anthropometrics.

**Objective:**

To explore the association between maternal PCOS and newborn anthropometrics and the modifying effects of maternal body mass index, PCOS phenotype, and gestational diabetes.

**Design, Setting, and Participants:**

This cohort study followed up women from the first half of pregnancy to birth and combined data from 3 clinical trials of pregnant women with PCOS and a reference population consisting of participants in the Norwegian Mother, Father, and Child Cohort (MoBa) Study, with data from the Medical Birth Registry of Norway. The recruitment period for the clinical trials was between October 1, 2000, and August 31, 2017, and for MoBa, between July 1, 1999, and December 31, 2008. Participants included women with singleton pregnancies and live-born children. Data were analyzed from January 1 to June 15, 2023.

**Exposure:**

Maternal PCOS status.

**Main Outcomes and Measures:**

Newborn birth weight, birth length, and head circumference as continuous variables and *z* scores, and ponderal index (calculated as the birth weight in grams × 100 divided by the birth length in centimeters cubed), placenta weight, and ratio of birth weight to placenta weight (BWPW).

**Results:**

The cohort included 390 pregnant women with PCOS (mean [SD] age, 29.6 [4.2] years) and 68 708 women in the reference group (mean [SD] age, 30.4 [4.5] years). Offspring in the PCOS group had lower birth weight, birth length, and head circumference than in the reference group offspring. The estimated mean differences in *z* scores were −0.26 (95% CI, −0.38 to −0.14) for birth weight, −0.19 (95% CI, −0.33 to −0.05) for birth length, and −0.13 (95% CI, −0.26 to −0.01) for head circumference. The PCOS group also had a lower ponderal index (−0.04 [95% CI, −0.07 to −0.004] g × 100/cm^3^). The association between growth restriction and PCOS was more apparent when additionally adjusting for body mass index. Neither PCOS phenotype nor gestational diabetes diagnosis was associated with neonatal anthropometry in women with PCOS.

**Conclusions and Relevance:**

In this cohort of mother-infant pairs, maternal PCOS status was associated with lower birth weight, shorter birth length, and smaller head circumference in the offspring. This growth restriction was more pronounced when adjusting for BMI, providing insight into the association between PCOS and body mass index. The study contributed to the understanding of how PCOS affects the offspring.

## Key Points

**Question:**

How is maternal polycystic ovary syndrome (PCOS) status associated with newborn anthropometrics?

**Findings:**

This cohort study compared 390 newborns of women with PCOS with 68 708 newborns of women in a reference population. An association was found between maternal PCOS status and growth restriction in the newborn, expressed as low birth weight, shorter birth length, and smaller head circumference.

**Meaning:**

These findings suggest that infants born to mother withs PCOS may have smaller anthropometrics than infants born to mothers in a reference population, an important contribution to the understanding of how PCOS affects the offspring.

## Teaser

This cohort study assesses the association of maternal polycystic ovary syndrome (PCOS) status with newborn anthropometrics and what modifying effect maternal body mass index, PCOS phenotype, or gestational diabetes may have.

## Introduction

Polycystic ovary syndrome (PCOS) is a common endocrine disorder estimated to affect 8% to 20% of women.^1,2^ PCOS is associated with poor metabolic health,^3^ reduced fertility, and increased risk of pregnancy complications such as gestational diabetes (GD), preeclampsia, and preterm birth.^4^ Hyperandrogenism, hyperinsulinemia, and early mobilization of cytokines with activated immune status are characteristics of PCOS that may influence the intrauterine environment, with a potential impact on fetal growth and offspring health.^5,6^ While some studies report lower birth weight in children born to women with PCOS compared with controls,^7,8,9,10^ others find no association between PCOS and the rates of small-for-gestational-age (SGA) or large-for-gestational-age (LGA) babies^11,12,13,14,15^ or find that associations are no longer apparent after adjusting for presumed confounding factors.^16,17,18^ The systematic reviews underpinning the 2023 International Evidence-Based Guideline for the Assessment and Management of Polycystic Ovary Syndrome identified a higher risk of low birth weight and intrauterine growth restriction among newborns of mothers with PCOS compared with controls.^2,19^

The primary aim of this study was to further explore the association of maternal PCOS with gestational age– and sex-adjusted birth anthropometrics by comparing children born to women with PCOS with a large reference population. The secondary aim was 3-fold: to explore the association of maternal PCOS with ponderal index, placenta weight, and birth weight to placenta weight (BWPW) ratio; to assess whether the association of PCOS with these measures was modified by maternal body mass index (BMI; calculated as the weight in kilograms divided by the height in meters squared); and to explore whether the presence of hyperandrogenic vs normoandrogenic PCOS phenotypes or GD moderates the association with neonatal anthropometrics.

## Methods

This cohort study combines data from 3 clinical trials of pregnant women with PCOS and a reference population consisting of participants in the Norwegian Mother, Father and Child Cohort (MoBa) Study. Women with PCOS were recruited from October 1, 2000, to August 31, 2017, while women included from the MoBa study were those with children born from January 1, 2000, to December 31, 2008. Both studies recruited women in the first half of pregnancy, and written informed consent was obtained from all participants. The studies were approved by the Regional Committee for Medical and Health Research Ethics. Data were analyzed from January 1 to June 15, 2023. The study was presented in accordance with the Strengthening the Reporting of Observational Studies in Epidemiology (STROBE) reporting guideline for cohort studies.

The PCOS group consisted of participants from the placebo groups of 3 randomized clinical trials comparing metformin treatment with placebo in women with PCOS: the Pilot study (postregistered),^20^ the Metformin in Pregnant PCOS Women (PregMet) study (NCT00159536),^21^ and the PregMet 2 study (NCT01587378).^22^ The Pilot study included 40 women at the University Hospital of Trondheim, Trondheim, Norway, of whom 22 were randomized to placebo. The PregMet and PregMet 2 studies were multicenter trials from hospitals in Norway, Sweden, and Iceland and included 274 and 487 pregnancies, respectively, with 138 and 243 randomized to placebo. All participants met the Rotterdam criteria for the PCOS diagnosis.^23^ Inclusion criteria were (1) PCOS diagnosis before pregnancy, (2) age between 18 and 45 years, (3) gestational age of 5 to 12 weeks, and (4) singleton viable fetus. We included 390 pregnancies in the present analyses after exclusion of early miscarriage (n = 3), late miscarriage (n = 8), and intrauterine fetal death (n = 2) (eFigure 1 in Supplement 1).

The MoBa Study is a population-based pregnancy cohort study conducted by the Norwegian Institute of Public Health. Participants were recruited from all over Norway from July 1, 1999, to December 31, 2008. Forty-one percent of the women consented to participate. The cohort included approximately 114 500 children, 95 200 mothers, and 75 200 fathers. The present study was based on version 12 of the quality-assured data files released for research in 2021. The establishment of the MoBa cohort and initial data collection was based on a license from the Norwegian Data Protection Agency and approval from the Regional Committees for Medical and Health Research Ethics. The MoBa cohort is currently regulated by the Norwegian Health Registry Act. The present study was approved by the Regional Committees for Medical and Health Research Ethics. The Medical Birth Registry of Norway (MBRN) is a national health registry containing information about all births in Norway. Material from the MoBa Study was used as reference population, supplemented with data from the MBRN.^24,25,26^

The reference population consisted of 73 758 mother-child dyads. Inclusion criteria consisted of (1) live-born children between 2000 and 2008 and (2) singletons. To match the PCOS population, we excluded mothers 18 years or younger or 45 years or older (n = 244).

Irregular menstruations were used as a proxy for PCOS, and 4109 women (5.6%) were excluded due to irregular or absent menstruations before pregnancy. Additional exclusions were 176 women due to gestational age of less than 23 plus 0 weeks or greater than 42 plus 6 weeks; 244 due to *z* scores for birth weight, birth length, or head circumference being greater than 5 SD over or below the mean, representing unrealistic measurements or extremely ill or affected neonates; and 277 due to unrealistic measurements of placenta weight (<100 g or >1630 g, which were greater than 5 SD over or below the mean). Finally, 68 708 reference pregnancies were analyzed (eFigure 1 in Supplement 1).

### End Points

Primary end points were birth weight, birth length, and head circumference as continuous variables and as *z* scores. The standard values of Niklasson and Albertsson-Wikland^27^ were used for the calculation of *z* scores, accounting for sex and gestational age of the newborn. Untrimmed placenta weight, ponderal index, and BWPW ratio were secondary end points. Ponderal index was calculated as the birth weight in grams × 100 divided by the birth length in centimeters cubed. We defined LGA and SGA as *z* scores greater than or less than 1.28, which correspond to 90th and 10th percentiles, respectively.

Neonatal measurements were performed according to hospital routine and obtained from medical records for the PCOS group and from the MBRN for the reference population. In cases of missing data, parental reports from the MoBa questionnaires were used.

### Covariates

We adjusted for the following potential confounding factors in the regression analyses: maternal age, parity, smoking, educational level, civil status, and maternal BMI. The covariates, baseline characteristics, and comorbidities were self-reported in both the PCOS group and reference population, except for parity and maternal BMI. Parity was self-reported in the PCOS group and obtained from the MBRN for the reference population. Maternal BMI for the PCOS group was measured by health care personnel at approximately 11 to 12 weeks of gestation; for the reference population, self-reported prepregnancy and current maternal weight were recorded via questionnaires at 15 weeks of gestation. To account for the difference in timing, the mean between the weights reported at the start of the pregnancy and week 15 was calculated for the reference participants. Maternal ethnicity was self-reported in the PCOS group, from predefined categories as Nordic and other (including African, Hispanic, Mediterranean, Middle Eastern, South and East Asian, and other). This information on the participants was important for the possibility to consider generalizability, as well as to consider potential confounding or mediating factors. Birth country for the reference population was self-reported without predefined options. Unrealistic measurements were removed using the following cutoffs: maternal weight of less than 40 kg and greater than 200 kg, maternal height of less than 145 cm and greater than 210 cm, and age at menarche of younger than 8 years and older than 20 years.

### Statistical Analysis

Statistical analyses were completed using Stata/MP, version 17 (StataCorp LLC). Maternal baseline characteristics were compared using the 2-tailed *t* test for continuous variables and χ*^2^* test for categorical variables. For the primary and secondary end points, crude and multivariable linear regression models were used to compare the newborns of women in the PCOS population with the newborns in the reference population. In model 1, we adjusted for the presumed confounding factors: maternal age, parity, smoking, educational level, and civil status. In model 2, we additionally adjusted for maternal BMI. We opted to include maternal BMI as an additional covariate in a separate analysis since it is unclear to what extent BMI is a consequence of PCOS and thus whether it is a confounder or a mediator of the association between PCOS and birth anthropometrics. In the analysis of placenta weight, ponderal index and BWPW ratio, sex, and gestational age were included as additional confounders in both models 1 and 2. In the analysis of *z* scores (for birth weight, birth length, and head circumference), sex, and gestational age were accounted for in the calculation of the *z* score and thus not included as covariates. The results are presented as estimated mean differences (MDs), together with 95% CIs. *P* < .05 was considered statistically significant. All tests were 2 sided. Apart from placenta weight, less than 2.5% of children had missing information for any of the neonatal measurements, as these were obtained from medical records or the national birth registry, although some observations were removed from the reference population because of extreme *z* score values. We have therefore not undertaken any additional analyses to address missing data. Placenta weight was also near complete for the reference group (67 057 of 68 708 [97.6%]); however, data were only available for 335 of 390 (85.9%) in the PCOS group. Nonetheless, we consider this amount missing to be sufficiently low.

In a separate regression analysis, we explored the influence of BMI on the association between PCOS and anthropometric measures by including an interaction term between BMI category and PCOS status. The World Health Organization classification of BMI was used to categorize BMI as underweight (BMI < 18.5), normal weight (BMI, 18.5-24.9), overweight (BMI, 25.0-29.9), and obesity (BMI ≥ 30.0).^28^ Participants with BMI of less than 18.5 were excluded due to small sample size.

Subsequently, we investigated potential differences in the neonatal anthropometric measures between subgroups of women with PCOS, including hyperandrogenic vs normoandrogenic PCOS phenotypes and those with and without GD. Both additional analyses were conducted in the PCOS population only, since information on the presence of GD was lacking for most of the women in the reference group. The hyperandrogenic phenotypes of PCOS included participants with both clinical and/or biochemical hyperandrogenism, in accordance with the Rotterdam criteria.^23^ Screening for GD was universal in the PCOS group. The Norwegian and Canadian criteria were used for GD diagnosis.^29^ The cutoff was fasting glucose values of greater than 95.5 mg/dL or 2-hour glucose values of 162.2 mg/dL or higher (to convert to mmol/L, multiply by 0.0555) during an oral glucose tolerance test.^29^

## Results

The present study included 390 mothers with PCOS and 68 708 mothers in the reference group. Mothers with PCOS were younger (mean [SD] age, 29.6 [4.2] vs 30.4 [4.5] years), had greater weight (mean [SD], 77.9 [17.5] vs 69.3 [12.3] kg), and shorter height (mean [SD], 167.2 [5.8] vs 168.2 [5.9] cm) than the mothers in the reference group (Table 1). The mean (SD) BMI in the PCOS group was 27.9 (6.4) vs 24.5 (4.1) in the reference group (*P* < .001), with a subsequently higher proportion of women with overweight or obesity. The populations were comparable in terms of age at menarche. The PCOS group had a higher proportion of reported comorbidities. The PCOS group was mainly Nordic (368 [94.4%]; data were not available for the reference group) (Table 1).

**Table 1.** Baseline Characteristics of Participants in the PCOS and Reference Populations

Characteristic	Reference group (n = 68 708)		PCOS group (n = 390)		*P* value
	No. of participants	Data^a^	No. of participants	Data^a^	
Age, mean (SD), y	68 708	30.4 (4.5)	390	29.6 (4.2)	<.001
Age at menarche, mean (SD), y	67 918	13.0 (1.3)	247	12.9 (1.6)	.36
Weight, mean (SD), kg	67 561	69.3 (12.3)	390	77.9 (17.5)	<.001
Height, mean (SD), cm	68 007	168.2 (5.9)	390	167.2 (5.8)	.001
BMI, mean (SD)	67 286	24.5 (4.1)	390	27.9 (6.4)	<.001
BMI category					
Underweight	67 286	945 (1.4)	390	2 (0.5)	<.001
Normal weight		42 311 (62.9)		148 (37.9)	
Overweight		17 463 (26.0)		116 (29.7)	
Obesity		6567 (9.8)		124 (31.8)	
Parity					
0	68 708	32 620 (47.5)	390	214 (54.9)	.004
≥1		36 088 (52.5)		176 (45.1)	
Mode of conception					
Spontaneous	NA	NA	390	213 (54.6)	NA
ART		NA		163 (41.8)	
Other		NA		14 (3.6)	
Educational level					
Elementary school	65 307	1460 (2.2)	254	10 (3.9)	.047
High school		21 209 (32.5)		67 (26.4)	
College		27 052 (41.4)		106 (41.7)	
University		15 586 (23.9)		71 (28.0)	
Civil status					
Married or cohabitant	68 708	66 129 (96.2)	255	248 (97.3)	.40
Other		2579 (3.8)		7 (2.7)	
Ancestry					
Nordic	NA	NA	390	368 (94.4)	NA
Other^b^		NA		22 (5.6)	
Birth country					
Norway or high-income countries	68 708	65 532 (95.4)	NA	NA	NA
Other		3176 (4.6)		NA	
PCOS phenotype					
HA plus OA plus PCOM	NA	NA	386	239 (61.3)	NA
HA plus OA		NA		21 (5.4)	
HA plus PCOM		NA		40 (10.3)	
OA plus PCOM		NA		86 (22.1)	
Eating disorder	68 708	1867 (2.7)	234	15 (6.4)	.001
Depression	68 708	4706 (6.8)	369	56 (15.2)	<.001
Asthma	68 708	5068 (7.4)	370	41 (11.1)	.007
Migraine	68 708	7606 (11.1)	370	60 (16.2)	.002
Anxiety	68 708	2550 (3.7)	136	2 (1.5)	.17
Hypertension	68 708	1076 (1.6)	368	13 (3.5)	.003
Hyperthyroidism or hypothyroidism	68 708	1891 (2.8)	370	29 (7.8)	<0.001

Abbreviations: ART, assisted reproductive technology; BMI, body mass index (calculated as the weight in kilograms divided by the height in meters squared); HA, hyperandrogenism; NA, not applicable or available; OA, oligoanovulation; PCOM, polycystic ovary morphology; PCOS, polycystic ovary syndrome.

^a^ Unless otherwise indicated, data are expressed as No. (%) of participants. Percentages have been rounded and may not total 100. Numbers vary owing to missing data.

^b^ Included African, Hispanic, Mediterranean, Middle Eastern, South and East Asian, and other.

Women with PCOS more often had preterm birth and cesarean section, and there was more preeclampsia in the PCOS group, although that result was not significant. Ninety-six women in the PCOS group (24.6%) developed GD (Table 2). Compared with the reference group, children born to women with PCOS had lower birth weight, birth length, and head circumference, both in observed values and corresponding *z* scores in the unadjusted analyses and adjusted models. In model 1, the estimated MDs in *z* scores were −0.26 (95% CI, −0.38 to −0.14) for birth weight, −0.19 (95% CI, −0.33 to −0.05) for birth length, and −0.13 (95% CI, −0.26 to −0.01) for head circumference. The estimated differences were greatest in model 2, after additionally adjusting for maternal BMI (MD for birth weight *z* score, −0.39 [95% CI, −0.51 to −0.27]; MD for birth length *z* score, −0.29 [95% CI, −0.44 to −0.15]; and MD for head circumference *z* score, −0.25 [95% CI, −0.37 to −0.12]) (Table 3).

**Table 2.** Baseline Data on Pregnancy and Delivery in the PCOS and Reference Populations

	Reference group (n = 68 708)		PCOS group (n = 390)		*P* value
	No. of participants	Data^a^	No. of participants	Data^a^	
Gestational diabetes	NA	NA	390	96 (24.6)	NA
Preeclampsia	68 708	2493 (3.6)	390	23 (5.9)	.017
Preterm birth	68 708	3147 (4.6)	390	32 (8.2)	.001
Mode of delivery					
Vaginal	54 711	47 135 (86.2)	390	315 (80.8)	.002
Cesarean	54 711	7576 (13.8)	390	75 (19.2)	
Offspring sex					
Boy	68 708	35 225 (51.3)	390	194 (49.7)	.55
Girl	68 708	33 483 (48.7)	390	196 (50.3)	
Gestational age, mean (SD), y	68 467	39.9 (1.8)	390	39.6 (2.1)	<.001
SGA, % (95% CI)^b^^,^^c^	5756	8.4 (8.2-8.6)	44	11.3 (8.5-14.8)	.04
AGA, % (95% CI)^b^^,^^d^	55 085	80.2 (79.9-80.5)	317	81.3 (77.1-84.9)	.58
LGA, % (95% CI)^b^^,^^e^	7789	11.3 (11.1-11.6)	29	7.4 (5.2-10.5)	.02

Abbreviations: AGA, appropriate for gestational age; LGA, large for gestational age; NA, not available; PCOS, polycystic ovary syndrome; SGA, small for gestational age.

^a^ Unless otherwise indicated, data are expressed as No. (%) of participants. Percentages have been rounded and may not total 100. Numbers vary owing to missing data.

^b^ Includes 68 630 observations in the reference group and 390 observations in the PCOS group.

^c^ Defined as *z* score for birth weight of less than −1.28.

^d^ Defined as *z* score for birth weight of −1.28 to 1.28.

^e^ Defined as *z* score for birth weight of greater than 1.28.

**Table 3.** Anthropometrics, Placenta Weight, Ponderal Index, and BWPW Ratio in the PCOS vs Reference Population

Covariate	Reference group (n = 68 708)		PCOS group (n = 390)		Crude model		Model 1^a^		Model 2^b^	
	No. of participants^c^	Mean (SD)	No. of participants^c^	Mean (SD)	MD (95% CI)	*P* value	MD (95% CI)	*P* value	MD (95% CI)	*P* value
Birth weight, g	68 696	3597 (554)	390	3 517 (600)	−80 (−135 to −25)	.005	−72 (−139 to −4)	.04	−133 (−200 to −66)	<.001
*z* Score	68 630	0.10 (1.01)	390	−0.12 (0.99)	−0.22 (−0.32 to −0.12)	<.001	−0.26 (−0.38 to −0.14)	<.001	−0.39 (−0.51 to −0.27)	<.001
Birth length, cm	67 519	50.4 (2.3)	383	50.1 (2.6)	−0.3 (−0.5 to −0.1)	.02	−0.2 (−0.5-0.1)	.24	−0.3 (−0.6 to −0.1)	.02
*z* Score	67 459	−0.23 (1.16)	383	−0.43 (1.14)	−0.20 (−0.32 to −0.09)	.001	−0.19 (−0.33 to −0.05)	.009	−0.29 (−0.44 to −0.15)	<.001
Head circumference, cm	67 453	35.3 (1.6)	386	35.1 (1.7)	−0.2 (−0.4 to −0.0)	.01	−0.1 (−0.3-0.1)	.23	−0.3 (−0.5 to −0.1)	.006
*z* Score	67 394	0.15 (1.03)	386	−0.00 (0.99)	−0.15 (−0.25 to −0.04)	.005	−0.13 (−0.26 to −0.01)	.04	−0.25 (−0.37 to −0.12)	<.001
Placenta										
Placenta weight, g	67 057	675 (147)	341	663 (147)	−11 (−27 to 4)	0.158	−8 (−26 to 11)^d^	0.410	−25 (−43 to −6)^d^	0.008
Ponderal index^e^	67 519	2.8 (0.3)	383	2.8 (0.3)	−0.02 (−0.05 to −0.002)	.07	−0.04 (−0.07 to −0.004)^d^	.03	−0.05 (−0.08 to −0.02)^d^	.001
BWPW ratio	67 046	5.5 (1.0)	341	5.4 (0.9)	0.1 (-0.2-0.0)	0.310	0.008 (−0.116 to −0.131)^d^	0.902	0.049 (−0.075 to 0.172)^d^	0.438

Abbreviations: BWPW, birth weight to placenta weight; MD, mean difference; PCOS, polycystic ovary syndrome.

^a^ Adjusted for maternal age, smoking, civil status, parity, and educational level.

^b^ Adjusted for maternal age, smoking, civil status, parity, educational level, and body mass index.

^c^ Numbers vary due to missing data.

^d^ Adjusted also for gestational age and offspring sex.

^e^ Calculated as the birth weight in grams × 100 divided by the birth length in centimeters cubed.

Mean (SD) placenta weight was found to be approximately the same in the unadjusted analysis and model 1, yet statistically significantly lower in model 2 (MD, −25 [95% CI, −43 to −6]). Ponderal index and BWPW ratio was similar in the two groups (Table 3). A higher proportion of offspring born to women in the reference group were LGA compared with offspring born to women with PCOS (7789 [11.3%; 95% CI, 11.1%-11.6%] vs 29 of 390 [7.4%; 95% CI, 5.2%-10.5%]; *P* = .02). More SGA offspring were born to mothers with PCOS than to mothers in the reference group (44 of 390 [11.3%; 95% CI, 8.5%-14.8%] vs 5756 of 68 467 [8.4%; 95% CI, 8.2%-8.6%]; *P* = .04) (Table 2).

In all BMI categories, birth weight, birth length, and head circumference were lower in neonates born to women with PCOS compared with women in the reference group, although these differences were not statistically significant for birth length or for head circumference in women with normal weight. The negative association between PCOS and birth anthropometrics was estimated to be progressively greater for children born to women with overweight and obesity; however, the degree of growth restriction was not statistically significantly different across BMI categories (Table 4 and Figure). Placenta weight was not different for the PCOS group among women of normal weight or overweight, but was statistically significantly lower for obese women. The BWPW ratio was approximately equal for the reference group and women with PCOS in all BMI categories (Table 4). Among women with PCOS, birth anthropometrics, placenta weight, ponderal index, and BWPW ratio were similar in women with hyperandrogenic versus normoandrogenic PCOS phenotypes (eTable 1 in Supplement 1), and in those with and without GD (eTable 2 in Supplement 1).

**Table 4.** Anthropometrics, Placenta Weight, Ponderal Index, and BWPW Ratio in the PCOS vs Reference Groups, by BMI Category^a^

Covariate	Maternal BMI 18.5-24.9					Maternal BMI 25.0-29.9					Maternal BMI ≥ 30.0					Interaction analysis, *P* value
	Reference group		PCOS group		MD (95% CI)	Reference group		PCOS group		MD (95% CI)	Reference group		PCOS group		MD (95% CI)	
	No. of participants	Mean (SD)	No. of participants	Mean (SD)		No. of participants	Mean (SD)	No. of participants	Mean (SD)		No. of participants	Mean (SD)	No. of participants	Mean (SD)		
Birth weight, g	42 304	3553 (527)	148	3474 (591)	−85 (−191 to 22)	17 459	3674 (567)	116	3561 (577)	−87 (−208 to 35)	6566	3716 (635)	124	3524 (637)	−188 (−310 to (−66)	.39
*z* Score	42 273	−0.00 (0.97)	148	−0.26 (0.93)	−0.33 (−0.52 to −0.14)	17 435	0.27 (1.01)	116	−0.05 (1.00)	−0.35 (−0.57 to −0.14)	6558	0.39 (1.13)	124	−0.01 (1.03)	−0.41 (−0.63 to −0.19)	.85
Birth length, cm	41 559	50.3 (2.3)	146	50.0 (2.6)	−0.1 (−0.5 to 0.4)	17 192	50.6 (2.4)	113	50.5 (2.4)	−0.1 (−0.6 to 0.5)	6451	50.7 (2.6)	122	49.8 (2.8)	−0.8 (−1.3 to −0.3)	.06
*z* Score	41 530	−0.31 (1.13)	146	−0.51 (1.18)	−0.16 (−0.39 to 0.06)	17 169	−0.09 (1.16)	113	−0.30 (1.17)	−0.23 (−0.49 to 0.03)	6445	−0.00 (1.24)	122	−0.46 (1.07)	−0.44 (−0.70 to −0.18)	.26
Head circumference, cm	41 646	35.2 (1.6)	148	35.1 (1.8)	−0.1 (−0.4 to 0.3)	17 086	35.5 (1.6)	115	35.1 (1.4)	−0.2 (−0.6 to 0.1)	6403	35.6 (1.7)	121	35.0 (1.9)	−0.5 (−0.9 to −0.1)	.19
*z S*core	41 618	0.05 (1.01)	148	−0.02 (1.01)	−0.12 (−0.32 to 0.08)	17 064	0.28 (1.03)	115	−0.05 (0.98)	−0.27 (−0.50 to −0.04)	6397	0.44 (1.10)	121	0.06 (0.96)	−0.32 (−0.56 to −0.09)	.38
Placenta																
Placenta weight, g	41 292	662 (141)	128	639 (141)	−23 (−52 to 6)^b^	17 022	693 (151)	98	682 (154)	−1 (−35 to 33)^b^	6432	713 (166)	113	676 (146)	−35 (−68 to −2)^b^	0.633
Ponderal index^c^	41 559	2.8 (0.3)	146	2.8 (0.3)	−0.06 (−0.11 to −0.01)^b^	17 192	2.8 (0.3)	113	2.8 (0.2)	−0.06 (−0.12 to −0.007)^b^	6451	2.8 (0.3)	122	2.8 (0.3)	−0.02 (−0.08 to 0.04)^b^	.49
BWPW ratio	41 285	5.5 (0.9)	128	5.6 (1.0)	0.1 (−0.1 to 0.3)^b^	17 019	5.4 (1.0)	98	5.4 (0.9)	−0.1 (−0.3 to 0.1)^b^	6431	5.4 (1.0)	113	5.3 (0.9)	0.1 (−0.1 to 0.3)^b^	0.483

Abbreviations: BMI, body mass index (calculated as the weight in kilograms divided by the height in meters squared); BWPW, birth weight to placenta weight; MD, mean difference; PCOS, polycystic ovary syndrome.

^a^ Mean difference, 95% CIs, and *P* values are calculated by interactional analysis with adjustment for maternal age, smoking, civil status, parity, and educational level. Numbers of participants vary due to missing data.

^b^ Adjusted also for gestational age and offspring sex.

^c^ Calculated as the birth weight in grams × 100 divided by the birth length in centimeters cubed.

**Figure.**
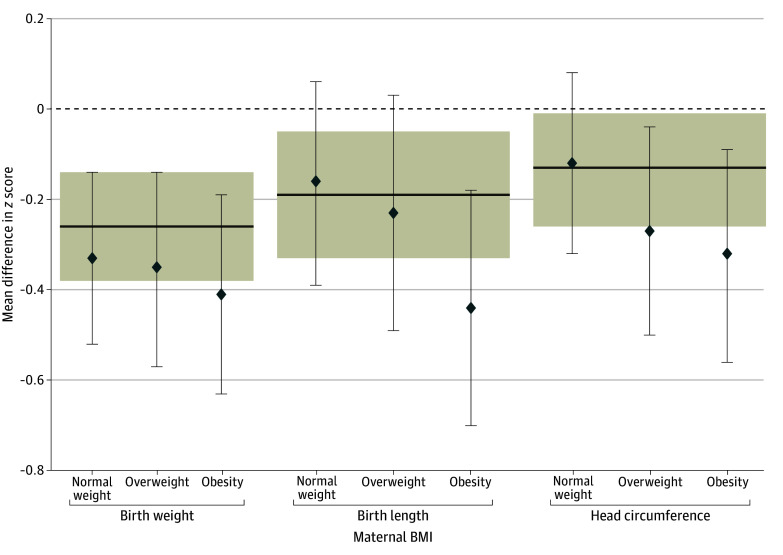
Association of Polycystic Ovary Syndrome (PCOS) With Birth Weight, Birth Length, and Head Circumference by Maternal Body Mass Index (BMI) Model is adjusted for maternal age, smoking, civil status, parity, and educational level. Normal weight is defined as a BMI (calculated as weight in kilograms divided by height in meters squared) of 18.5 to 24.9; overweight, 25.0 to 29.9; and obesity, 30.0 or greater. The shaded sections represent mean difference and 95% CI for regression analysis on *z* score anthropometrics in the PCOS vs reference groups. Dashed line (0) indicates no difference. The degree of growth restriction was not statistically significantly different between BMI subgroups (*P* = .85 for interaction for birth weight, *P* = .26 for interaction for birth length, and *P* = .38 for interaction for head circumference).

## Discussion

To our knowledge, this cohort study of the associations among PCOS status, maternal BMI subgroups, and neonate anthropometrics is unique. The main findings are that offspring of mothers with PCOS have reduced birth weight, birth length, and head circumference compared with the reference population. The growth restriction observed increased with adjustment for BMI (model 2). Placenta weight was lower in women with PCOS once adjusting for BMI, yet the BWPW ratio remained similar indicating a compromised but adequately efficient placenta for birthweight.

PCOS status was associated with a reduction in all neonatal anthropometric measures, becoming more apparent with increased maternal overweight. With higher maternal BMI, greater gestational weight gain, and more GD in women with PCOS,^4,11,30^ one would expect larger offspring compared with the reference population. Interestingly, previous studies have found the opposite association.^7,8,9,19^ In the current analyses, the reduction in birth anthropometrics was most pronounced in model 2, which additionally adjusted for maternal BMI. This indicates an association between PCOS and growth restriction that may be partially camouflaged or counteracted by higher maternal BMI. The growth restrictive mechanism of PCOS appears to be stronger than the anabolic drive by excess maternal weight, higher gestational weight gain, and high incidence of GD otherwise seen in women with PCOS. These findings are in line with and extend the understanding of fetal growth restriction stated in the 2023 International Evidence-Based Guideline for the Assessment and Management of Polycystic Ovary Syndrome.^2,19^

The growth restriction observed in offspring in the PCOS group was most pronounced for birth weight and less so for birth length and head circumference. This finding may indicate brain sparing, a fetal prioritization of blood flow to the brain to protect growth and development, in line with states of starvation or placental insufficiency.^31,32^ Additionally, the growth restriction appeared to be more pronounced among women with overweight and obesity. Overweight and obesity aggravate most symptoms of PCOS,^33,34^ and the more pronounced growth restriction seen in these groups may represent a more severe PCOS. Stokkeland et al^6^ previously reported that women with PCOS showed marked changes in serum cytokine levels throughout pregnancy compared with controls. High immunological activation in the first and third trimesters and inflammatory cytokine levels contributed strongly to these patterns. Members of our study group found that body weight appears to modulate the immune system in PCOS, with elevated levels of numerous multifunctional cytokines in women with overweight and obesity.^6^ Possibly, the more activated immune state seen in PCOS, and particularly among women with overweight or obesity combined with PCOS, may partially account for the growth restriction demonstrated in PCOS offspring.

### Potential Mechanisms for Growth Restrictions

#### Androgens

Maternal and/or intrauterine hyperandrogenism has been suggested as a potential mechanism for growth restriction.^9,35,36^ In contrast to these studies, we found no conclusive difference in neonatal anthropometrics between the women with normoandrogenic and hyperandrogenic phenotypes of PCOS. Other studies^37,38,39,40^ in line with ours found no difference in the prevalence of SGA offspring in hyperandrogenic vs normoandrogenic PCOS phenotypes.

#### Placenta

Animal models of PCOS indicate placenta dysfunction as a possible cause of growth restriction.^41,42^ In the present study, mothers with PCOS more often had newborns with SGA and less often with LGA, and lower placenta weight once adjusting for confounding factors including BMI and gestational age. However, there was no apparent difference between the BWPW ratio among women with PCOS compared with the reference population. The BWPW ratio is a proxy for placental efficacy, and the lack of difference in BWPW suggests that the placenta of women with PCOS is normally efficient relative to birthweight.^43^

Several studies^44,45,46^ find that low birth weight generally is associated with cardiovascular diseases and type 2 diabetes later in life. Seventy children born to mothers with PCOS in the PregMet study^8^ were followed up at 8 years of age. These children had more central obesity compared to a reference population, which indicates that the present findings of restricted growth and smaller placentas is clinically meaningful and seem to be a marker of long-term metabolic inferiority. Notably, the growth discrepancy at birth was largest in neonates of mothers with obesity, in line with the understanding of maternal obesity being more detrimental for the long-term health of offspring.^47,48,49,50^ Smaller head circumference in neonates in the PCOS group might influence cognitive neuropsychological function later in life. However, the follow-up with cognitive testing of a limited number of children from the PregMet study showed intellectual functioning in the reference range.^51^ Large register studies and metanalyses^52,53,54^ report a higher prevalence of attention-deficit disorders, autism spectrum disorder, and chronic tic disorder among offspring of women with PCOS.

### Strengths and Limitations

A major strength of the study is its large sample size and the possibility of reporting *z* scores, giving a precise estimate in which we account for offspring sex and gestational age. Possible limitations include the following: (1) data on maternal weight and height in the PCOS group are measured, while they are self-reported in the reference population; and (2) irregular menstruation as a proxy for PCOS in the reference population identifies only a proportion of the women with PCOS. This results in an underestimation of the observed differences. We cannot exclude selection bias of study participants. Participants are mainly of Nordic ancestry, so the results may not be directly applicable to all ancestries. The Norwegian and Canadian GD criteria used in this study limit the generalizability.

## Conclusions

The novel finding of the present cohort study is that neonates of mothers with PCOS appear to be growth restricted, with lower birth weight, birth length, and head circumference. This growth restriction also appeared to be more pronounced when adjusting for BMI and potentially for women with overweight and obesity, although the modifying effect of maternal BMI will need to be confirmed in future studies. Further studies on this subject are needed.

## Notes

Supplement 1. **eTable 1.** Anthropometrics, Placenta Weight, Ponderal Index, and Birth Weight to Placenta Weight Ratio: Hyperandrogenic vs Normoandrogenic PCOS Phenotype **eTable 2.** Anthropometrics, Placenta Weight, Ponderal Index, and Birth Weight to Placenta Weight Ratio: PCOS Pregnancies With and Without GD **eFigure.** Flowchart on Inclusion and Exclusion

Supplement 2.
Data Sharing Statement

## References

[1] Deswal R, Narwal V, Dang A, Pundir CS. The prevalence of polycystic ovary syndrome: a brief systematic review. J Hum Reprod Sci. 2020;13(4):261-271. doi:10.4103/jhrs.JHRS_95_18 33627974 PMC7879843

[2] Teede HJ, Tay CT, Laven JJE, . Recommendations from the 2023 International Evidence-Based Guideline for the Assessment and Management of Polycystic Ovary Syndrome. J Clin Endocrinol Metab. 2023;108(10):2447-2469. doi:10.1210/clinem/dgad463 37580314 PMC10505534

[3] Dumesic DA, Oberfield SE, Stener-Victorin E, Marshall JC, Laven JS, Legro RS. Scientific statement on the diagnostic criteria, epidemiology, pathophysiology, and molecular genetics of polycystic ovary syndrome. Endocr Rev. 2015;36(5):487-525. doi:10.1210/er.2015-1018 26426951 PMC4591526

[4] Kjerulff LE, Sanchez-Ramos L, Duffy D. Pregnancy outcomes in women with polycystic ovary syndrome: a metaanalysis. Am J Obstet Gynecol. 2011;204(6):558.e1-558.e6. doi:10.1016/j.ajog.2011.03.021 21752757

[5] Palomba S, de Wilde MA, Falbo A, Koster MP, La Sala GB, Fauser BC. Pregnancy complications in women with polycystic ovary syndrome. Hum Reprod Update. 2015;21(5):575-592. doi:10.1093/humupd/dmv029 26117684

[6] Stokkeland LMT, Giskeødegård GF, Ryssdal M, . Changes in serum cytokines throughout pregnancy in women with polycystic ovary syndrome. J Clin Endocrinol Metab. 2022;107(1):39-52. doi:10.1210/clinem/dgab684 34529073 PMC8684459

[7] Mann A, Sagili H, Subbaiah M. Pregnancy outcome in women with polycystic ovary syndrome. J Obstet Gynaecol India. 2020;70(5):360-365. doi:10.1007/s13224-020-01356-y 33041553 PMC7515985

[8] Fornes R, Simin J, Nguyen MH, . Pregnancy, perinatal and childhood outcomes in women with and without polycystic ovary syndrome and metformin during pregnancy: a nationwide population-based study. Reprod Biol Endocrinol. 2022;20(1):30. doi:10.1186/s12958-022-00905-6 35130922 PMC8819934

[9] Mehrabian F, Kelishadi R. Comparison of the metabolic parameters and androgen level of umbilical cord blood in newborns of mothers with polycystic ovary syndrome and controls. J Res Med Sci. 2012;17(3):207-211. 23267369 PMC3527035

[10] Hjorth-Hansen A, Salvesen Ø, Engen Hanem LG, . Fetal growth and birth anthropometrics in metformin-exposed offspring born to mothers with PCOS. J Clin Endocrinol Metab. 2018;103(2):740-747. doi:10.1210/jc.2017-01191 29165598

[11] Kent J, Dodson WC, Kunselman A, ; Reproductive Medicine Network. Gestational weight gain in women with polycystic ovary syndrome: a controlled study. J Clin Endocrinol Metab. 2018;103(11):4315-4323. doi:10.1210/jc.2017-02764 30085187 PMC6194806

[12] Li Y, Ruan X, Wang H, . Comparing the risk of adverse pregnancy outcomes of Chinese patients with polycystic ovary syndrome with and without antiandrogenic pretreatment. Fertil Steril. 2018;109(4):720-727. doi:10.1016/j.fertnstert.2017.12.023 29525688

[13] Christ JP, Gunning MN, Meun C, . Pre-conception characteristics predict obstetrical and neonatal outcomes in women with polycystic ovary syndrome. J Clin Endocrinol Metab. 2019;104(3):809-818. doi:10.1210/jc.2018-01787 30590587

[14] Mills G, Badeghiesh A, Suarthana E, Baghlaf H, Dahan MH. Associations between polycystic ovary syndrome and adverse obstetric and neonatal outcomes: a population study of 9.1 million births. Hum Reprod. 2020;35(8):1914-1921. doi:10.1093/humrep/deaa144 32644124

[15] Wang Y, Guo L, Jiang J, Wang F, Hardiman PJ, Qu F. Development of 1-2 years offspring born to mothers with polycystic ovary syndrome. J Coll Physicians Surg Pak. 2021;31(10):1186-1190. doi:10.29271/jcpsp.2021.10.1186 34601839

[16] Subramanian A, Lee SI, Phillips K, . Polycystic ovary syndrome and risk of adverse obstetric outcomes: a retrospective population-based matched cohort study in England. BMC Med. 2022;20(1):298. doi:10.1186/s12916-022-02473-3 36038914 PMC9425992

[17] Farland LV, Stern JE, Liu CL, . Polycystic ovary syndrome and risk of adverse pregnancy outcomes: a registry linkage study from Massachusetts. Hum Reprod. 2022;37(11):2690-2699. doi:10.1093/humrep/deac210 36149255 PMC9627555

[18] Finnbogadóttir SK, Glintborg D, Jensen TK, Kyhl HB, Nohr EA, Andersen M. Insulin resistance in pregnant women with and without polycystic ovary syndrome, and measures of body composition in offspring at birth and three years of age. Acta Obstet Gynecol Scand. 2017;96(11):1307-1314. doi:10.1111/aogs.13200 28804876

[19] Teede H, Tay CT, Laven JSE, . International evidence-based guideline for the assessment and management of polycystic ovary syndrome 2023. Monash University. Updated February 2023. Accessed April 2, 2024. https://www.monash.edu/__data/assets/pdf_file/0003/3379521/Evidence-Based-Guidelines-2023.pdf

[20] Vanky E, Salvesen KA, Heimstad R, Fougner KJ, Romundstad P, Carlsen SM. Metformin reduces pregnancy complications without affecting androgen levels in pregnant polycystic ovary syndrome women: results of a randomized study. Hum Reprod. 2004;19(8):1734-1740. doi:10.1093/humrep/deh347 15178665

[21] Vanky E, Stridsklev S, Heimstad R, . Metformin versus placebo from first trimester to delivery in polycystic ovary syndrome: a randomized, controlled multicenter study. J Clin Endocrinol Metab. 2010;95(12):E448-E455. doi:10.1210/jc.2010-0853 20926533

[22] Løvvik TS, Carlsen SM, Salvesen Ø, . Use of metformin to treat pregnant women with polycystic ovary syndrome (PregMet2): a randomised, double-blind, placebo-controlled trial. Lancet Diabetes Endocrinol. 2019;7(4):256-266. doi:10.1016/S2213-8587(19)30002-6 30792154

[23] Rotterdam ESHRE/ASRM-Sponsored PCOS consensus workshop group. Revised 2003 consensus on diagnostic criteria and long-term health risks related to polycystic ovary syndrome (PCOS). Hum Reprod. 2004;19(1):41-47. doi:10.1093/humrep/deh098 14688154

[24] Magnus P, Birke C, Vejrup K, . Cohort profile update: the Norwegian Mother and Child Cohort Study (MoBa). Int J Epidemiol. 2016;45(2):382-388. doi:10.1093/ije/dyw029 27063603

[25] Magnus P, Irgens LM, Haug K, Nystad W, Skjaerven R, Stoltenberg C; MoBa Study Group. Cohort profile: the Norwegian Mother and Child Cohort Study (MoBa). Int J Epidemiol. 2006;35(5):1146-1150. doi:10.1093/ije/dyl170 16926217

[26] Irgens LM. The Medical Birth Registry of Norway: epidemiological research and surveillance throughout 30 years. Acta Obstet Gynecol Scand. 2000;79(6):435-439. doi:10.1034/j.1600-0412.2000.079006435.x 10857866

[27] Niklasson A, Albertsson-Wikland K. Continuous growth reference from 24th week of gestation to 24 months by gender. BMC Pediatr. 2008;8:8. doi:10.1186/1471-2431-8-8 18307822 PMC2294116

[28] World Health Organization. Physical status: the use and interpretation of anthropometry: report of a WHO expert committee. February 28, 1995. Accessed March 26, 2024. https://iris.who.int/handle/10665/37003 8594834

[29] Helsedirektoratet. Gestational diabetes: national professional guideline. Updated June 6, 2023. Accessed March 26, 2024. https://www.helsedirektoratet.no/retningslinjer/svangerskapsdiabetes

[30] Bahri Khomami M, Joham AE, Boyle JA, . The role of maternal obesity in infant outcomes in polycystic ovary syndrome—a systematic review, meta-analysis, and meta-regression. Obes Rev. 2019;20(6):842-858. doi:10.1111/obr.12832 30785659

[31] Roza SJ, Steegers EA, Verburg BO, . What is spared by fetal brain-sparing? fetal circulatory redistribution and behavioral problems in the general population. Am J Epidemiol. 2008;168(10):1145-1152. doi:10.1093/aje/kwn233 18826969

[32] Baschat AA. Fetal responses to placental insufficiency: an update. BJOG. 2004;111(10):1031-1041. doi:10.1111/j.1471-0528.2004.00273.x 15383103

[33] Pasquali R. Obesity and androgens: facts and perspectives. Fertil Steril. 2006;85(5):1319-1340. doi:10.1016/j.fertnstert.2005.10.054 16647374

[34] Ehrmann DA, Liljenquist DR, Kasza K, Azziz R, Legro RS, Ghazzi MN; PCOS/Troglitazone Study Group. Prevalence and predictors of the metabolic syndrome in women with polycystic ovary syndrome. J Clin Endocrinol Metab. 2006;91(1):48-53. doi:10.1210/jc.2005-1329 16249284

[35] Huang G, Aroner SA, Bay CP, . Sex-dependent associations of maternal androgen levels with offspring BMI and weight trajectory from birth to early childhood. J Endocrinol Invest. 2021;44(4):851-863. doi:10.1007/s40618-020-01385-4 32776198 PMC7873156

[36] de Wilde MA, Lamain-de Ruiter M, Veltman-Verhulst SM, . Increased rates of complications in singleton pregnancies of women previously diagnosed with polycystic ovary syndrome predominantly in the hyperandrogenic phenotype. Fertil Steril. 2017;108(2):333-340. doi:10.1016/j.fertnstert.2017.06.015 28778282

[37] Naver KV, Grinsted J, Larsen SO, . Increased risk of preterm delivery and pre-eclampsia in women with polycystic ovary syndrome and hyperandrogenaemia. BJOG. 2014;121(5):575-581. doi:10.1111/1471-0528.12558 24418062

[38] Mumm H, Jensen DM, Sørensen JA, . Hyperandrogenism and phenotypes of polycystic ovary syndrome are not associated with differences in obstetric outcomes. Acta Obstet Gynecol Scand. 2015;94(2):204-211. doi:10.1111/aogs.12545 25417943

[39] Dehghani Firoozabadi A, Dehghani Firouzabadi R, Eftekhar M, Sadat Tabatabaei Bafghi A, Shamsi F. Maternal and neonatal outcomes among pregnant women with different polycystic ovary syndrome phenotypes: a cross-sectional study. Int J Reprod Biomed. 2020;18(5):339-346. doi:10.18502/ijrm.v13i5.7154 32637862 PMC7306059

[40] Wei DM, Zhang ZZ, Wang Z, . Effect of hyperandrogenism on obstetric complications of singleton pregnancy from in vitro fertilization in women with polycystic ovary syndrome. Article in Chinese. Zhonghua Fu Chan Ke Za Zhi. 2018;53(1):18-22. 29374881 10.3760/cma.j.issn.0529-567X.2018.01.005

[41] Beckett EM, Astapova O, Steckler TL, Veiga-Lopez A, Padmanabhan V. Developmental programing: impact of testosterone on placental differentiation. Reproduction. 2014;148(2):199-209. doi:10.1530/REP-14-0055 24840528 PMC4091887

[42] Manti M, Pui HP, Edström S, . Excess of ovarian nerve growth factor impairs embryonic development and causes reproductive and metabolic dysfunction in adult female mice. FASEB J. 2020;34(11):14440-14457. doi:10.1096/fj.202001060R 32892421

[43] Hayward CE, Lean S, Sibley CP, . Placental adaptation: what can we learn from birthweight:placental weight ratio? Front Physiol. 2016;7:28. doi:10.3389/fphys.2016.00028 26903878 PMC4742558

[44] Hales CN, Barker DJ, Clark PM, . Fetal and infant growth and impaired glucose tolerance at age 64. BMJ. 1991;303(6809):1019-1022. doi:10.1136/bmj.303.6809.1019 1954451 PMC1671766

[45] Leon DA, Lithell HO, Vâgerö D, . Reduced fetal growth rate and increased risk of death from ischaemic heart disease: cohort study of 15 000 Swedish men and women born 1915-29. BMJ. 1998;317(7153):241-245. doi:10.1136/bmj.317.7153.241 9677213 PMC28614

[46] Forsén T, Eriksson J, Tuomilehto J, Reunanen A, Osmond C, Barker D. The fetal and childhood growth of persons who develop type 2 diabetes. Ann Intern Med. 2000;133(3):176-182. doi:10.7326/0003-4819-133-3-200008010-00008 10906831

[47] Chandrasekaran S, Neal-Perry G. Long-term consequences of obesity on female fertility and the health of the offspring. Curr Opin Obstet Gynecol. 2017;29(3):180-187. doi:10.1097/GCO.0000000000000364 28448277 PMC5983896

[48] Chiavaroli V, Hopkins SA, Biggs JB, . The associations between maternal BMI and gestational weight gain and health outcomes in offspring at age 1 and 7 years. Sci Rep. 2021;11(1):20865. doi:10.1038/s41598-021-99869-7 34675369 PMC8531053

[49] Lahti-Pulkkinen M, Bhattacharya S, Wild SH, . Consequences of being overweight or obese during pregnancy on diabetes in the offspring: a record linkage study in Aberdeen, Scotland. Diabetologia. 2019;62(8):1412-1419. doi:10.1007/s00125-019-4891-4 31214738 PMC6647186

[50] Turunen R, Pulakka A, Metsälä J, . Maternal diabetes and overweight and congenital heart defects in offspring. JAMA Netw Open. 2024;7(1):e2350579. doi:10.1001/jamanetworkopen.2023.50579 38180757 PMC10770771

[51] Greger HK, Hanem LGE, Østgård HF, Vanky E. Cognitive function in metformin exposed children, born to mothers with PCOS—follow-up of an RCT. BMC Pediatr. 2020;20(1):60. doi:10.1186/s12887-020-1960-2 32039724 PMC7008569

[52] Palm CVB, Glintborg D, Find LG, . Prenatal androgen exposure and traits of autism spectrum disorder in the offspring: Odense Child Cohort. J Autism Dev Disord. 2023;53(3):1053-1065. doi:10.1007/s10803-022-05446-w 35124780

[53] Cesta CE, Öberg AS, Ibrahimson A, . Maternal polycystic ovary syndrome and risk of neuropsychiatric disorders in offspring: prenatal androgen exposure or genetic confounding? Psychol Med. 2020;50(4):616-624. doi:10.1017/S0033291719000424 30857571 PMC7093321

[54] Katsigianni M, Karageorgiou V, Lambrinoudaki I, Siristatidis C. Maternal polycystic ovarian syndrome in autism spectrum disorder: a systematic review and meta-analysis. Mol Psychiatry. 2019;24(12):1787-1797. doi:10.1038/s41380-019-0398-0 30867561

