# Errors in Abstract, Tables, Results, Discussion, and Supplement 1

**DOI:** 10.1001/jamanetworkopen.2026.2845

**Published:** 2026-02-25

In the Original Investigation titled “Growth Restriction in the Offspring of Mothers With Polycystic Ovary Syndrome,”^1^ published August 27, 2024, there were errors in the Abstract, Results, Discussion, and Supplement 1. In the Abstract results, there should not have been references to differences in placenta weight or ratio of birth weight to placenta weight (BWPW). In Table 1, there were errors in proportion of women with different polycystic ovary syndrome (PCOS) phenotype categories, and hyperthyroidism or hypothyroidism. In Table 2, there were errors in the proportion with preeclampsia. In Table 3, there were errors in the results for placenta weight and BWPW ratio. In Table 4, there were errors in results for placenta weight and BWPW ratio. In the Results, updates were required in the third paragraph to reflect the findings with corrected placenta weight and BWPW ratio; specifically, placenta weight was only statistically significantly lower after adjusting for confounding factors and body mass index (BMI) and BWPW was no longer found to differ between groups; and updates were required in the fourth paragraph of Results to reflect that placenta weight was only lower among obese women with PCOS, and BWPW ratio was approximately equal for the reference group and women with PCOS in all BMI categories. In the first paragraph of the Discussion, updates were required to revise the description of difference in placenta weight and lack of difference in BWPW ratio; in the fifth paragraph (under the subhead *Placenta*), much of this discussion stemmed from the finding that the BWPW-ratio was higher among women with PCOS, but this finding is no longer observed with the corrected data; in the eighth paragraph (under *Conclusions*), updates were required to remove the sentence about placenta weight and BWPW. In Supplement 1, the entire eTable 1 required correction due to correction in classification of PCOS phenotype, in addition to corrections in placenta weight and BWPW ratio; in eTable 2, updates were required of results for placenta weight and BWPW ratio. This article has been corrected.^1^
