# Supplementary material for: Growth Restriction in the Offspring of Mothers With Polycystic Ovary Syndrome
Source: JAMA Netw Open. 2024 Aug 27;7(8):e2430543. doi: 10.1001/jamanetworkopen.2024.30543 (PMC11350484; doi:10.1001/jamanetworkopen.2024.30543)
Supplement: Supplement 1. — eTable 1. Anthropometrics, Placenta Weight, Ponderal Index, and Birth Weight to Placenta Weight Ratio: Hyperandrogenic vs Normoandrogenic PCOS Phenotype eTable 2. Anthropometrics, Placenta Weight, Ponderal Index, and Birth Weight to Placenta Weight Ratio: PCOS Pregnancies With and Without GD eFigure. Flowchart on Inclusion and Exclusion [file jamanetwopen-e2430543-s001.pdf]

Supplementary Online Content

Talmo MSA, Fløysand IS, Nilsen GØ, et al. Growth restriction in the offspring of mothers with polycystic ovary syndrome. *JAMA Netw Open*. 2024;7(8):e2430543. doi:10.1001/jamanetworkopen.2024.30543

eTable 1. Anthropometrics, Placenta Weight, Ponderal Index, and Birth Weight to Placenta Weight Ratio: Hyperandrogenic vs Normoandrogenic PCOS Phenotype

eTable 2. Anthropometrics, Placenta Weight, Ponderal Index, and Birth Weight to Placenta Weight Ratio: PCOS Pregnancies With and Without GD

eFigure. Flowchart on Inclusion and Exclusion

This supplementary material has been provided by the authors to give readers additional information about their work.

© 2024 Talmo MSA et al. *JAMA Network Open*.

eTable 1. Anthropometrics, Placenta Weight, Ponderal Index, and Birth Weight to Placenta Weight Ratio:

Hyperandrogenic vs Normoandrogenic PCOS Phenotype

	**Hyper-androgenic**		**Normo-androgenic**		**Not Adjusted**		**Model 1[a]**		**Model 2[b]**	
	n[c]	*Mean (SD)*	*n[c]*	*Mean (SD)*	*Mean difference (95% CI)*	*p-value*	*Mean difference (95% CI)*	*p-value*	*Mean difference (95% CI)*	*p-value*
**Birthweight**										
In grams	300	3 545 (590)	86	3 443 (595)	-102 (-244 -40)	0.160	23 (-148-194)	0.788	21 (-153-195)	0.813
Z-score	300	-0.11 (0.99)	86	-0.09 (0.91)	0.02 (-0.21-0.26)	0.859	0.08 (-0.23-0.38)	0.617	0.13 (-0.18-0.43)	0.414
**Birth length**										
In cm	294	50.2 (2.5)	85	49.8 (2.9)	-0.4 (-1.0-0.3)	0.240	0.3 (-0.5-1.2)	0.395	0.2 (-0.6-1.1)	0.558
Z-score	294	-0.43 (1.15)	85	-0.37 (1.07)	0.06 (-0.22-0.33)	0.690	0.22 (-0.15-0.59)	0.239	0.23 (-0.15-0.60)	0.237
**Head circumference**										
In cm	296	35.1 (1.7)	86	34.9 (1.6)	-0.2 (-0.6-0.2)	0.299	0.2 (-0.3-0.7)	0.408	0.2 (-0.3-0.7)	0.386
Z-score	296	-0.01 (0.98)	86	0.05 (1.00)	0.06 (-0.18-0.30)	0.626	0.15 (-0.16-0.46)	0.337	0.21 (-0.10-0.52)	0.192
**Placenta weight (g)**	263	672 (142)	77	633 (163)	-39 (-77-(-2))	0.040	8 (-35-52)[d]	0.699	15 (-29-59)[d]	0.496
**PI (grams x100/cm^3)**	294	2.8 (0.3)	85	2.8 (0.2)	-0.0 (-0.1-0.0)	0.351	-0.0 (-0.1-0.1)[d]	0.487	-0.0 (-0.1-0.1)[d]	0.757
**BWPW-ratio (bw/pw)**	263	5.4 (0.9)	77	5.5 (0.9)	0.2 (-0.1 -0.4)	0.201	-0.0 (-0.3 -0.3)[d]	0.951	-0.0 (-0.3-0.3)[d]	0.855

BMI: body mass index; bw: birthweight; BWPW-ratio: birthweight-to-placenta-weight ratio; CI: confidence interval; n: number; g: grams; GDM: gestational diabetes mellitus;

PCOS: polycystic ovary syndrome; PI: ponderal index; pw: placenta weight; SD: standard deviation.

[a] Model 1: adjusted for maternal age, smoking, civil status, parity, and educational level.

[b] Model 2: adjusted for maternal age, smoking, civil status, parity, educational level and BMI.

[c] Numbers vary due to missing data and excluded participants.

[d] Adjustment also for gestational age and offspring sex.

© 2024 Talmo MSA et al. *JAMA Network Open*.

eTable 2. Anthropometrics, Placenta Weight, Ponderal Index, and Birth Weight to Placenta Weight Ratio: PCOS Pregnancies With and Without GD

	**Non-GDM**		**GDM**		**Not Adjusted**		**Model 1[a]**		**Model 2[b]**	
	n[c]	*Mean (SD)*	*n[c]*	*Mean (SD)*	*Mean difference (95% CI)*	*p-value*	*Mean difference (95% CI)*	*p-value*	*Mean difference (95% CI)*	*p-value*
**Birthweight**										
In grams	294	3 556 (596)	96	3 397 (600)	-159 (-297-(-21))	0.024	-102 (-261-56)	0.206	-101 (-263-61)	0.221
Z-score	294	-0.10 (0.94)	96	-0.17 (1.11)	-0.07 (-0.30-0.16)	0.549	0.03 (-0.25-0.31)	0.812	-0.01 (-0.30-0.27)	0.928
**Birth length**										
In cm	289	50.3 (2.7)	94	49.6 (2.5)	-0.6 (-1.2-(-0.0))	0.047	-0.2 (-0.9-0.6)	0.614	-0.1 (-0.8-0.7)	0.860
Z-score	289	-0.42 (1.13)	94	-0.45 (1.19)	-0.03 (-0.30-0.24)	0.819	0.17 (-0.17-0.50)	0.338	0.17 (-0.17-0.52)	0.324
**Head circumference**										
In cm	291	35.2 (1.7)	95	34.9 (1.7)	-0.3 (-0.7-0.1)	0.153	-0.3 (-0.7 - 0.2)	0.273	-0.3 (-0.7-0.2)	0.263
Z-score	291	-0.00 (0.94)	95	0.01 (1.12)	0.01 (-0.22-0.24)	0.920	-0.05 (-0.33-0.24)	0.752	-0.10 (-0.39-0.19)	0.488
**Placenta weight (g)**	255	664 (145)	86	661 (154)	-4 (-40-32)	0.834	15 (-24-55)[d]	0.446	9 (-31-49)[d]	0.662
**PI (grams x100/cm^3)**	289	2.8 (0.2)	94	2.8 (0.3)	-0.0 (-0.1-0.0)	0.496	-0.0 (-0.1-0.1)[d]	0.618	-0.0 (-0.1-0.0)[d]	0.352
**BWPW-ratio (bw/pw)**	255	5.5 (0.9)	86	5.3 (0.9)	-0.2 (-0.4-0.0)	0.065	-0.1 (-0.4-0.1)[d]	0.369	-0.1 (-0.4-0.2)[d]	0.438

BMI: body mass index; bw: birthweight; BWPW-ratio: birthweight-to-placenta-weight ratio; CI: confidence interval; g: grams; n: number; PCOS: polycystic ovary syndrome;

PI: ponderal index; pw: placenta weight; SD: standard deviation.

[a] Model 1: adjusted for maternal age, smoking, civil status, parity, and educational level.

[b] Model 2: adjusted for maternal age, smoking, civil status, parity, educational level and BMI.

[c] Numbers vary due to missing data and excluded participants.

[d] Adjustment also for gestational age and offspring sex.

© 2024 Talmo MSA et al. *JAMA Network Open*.

eFigure. Flowchart on Inclusion and Exclusion

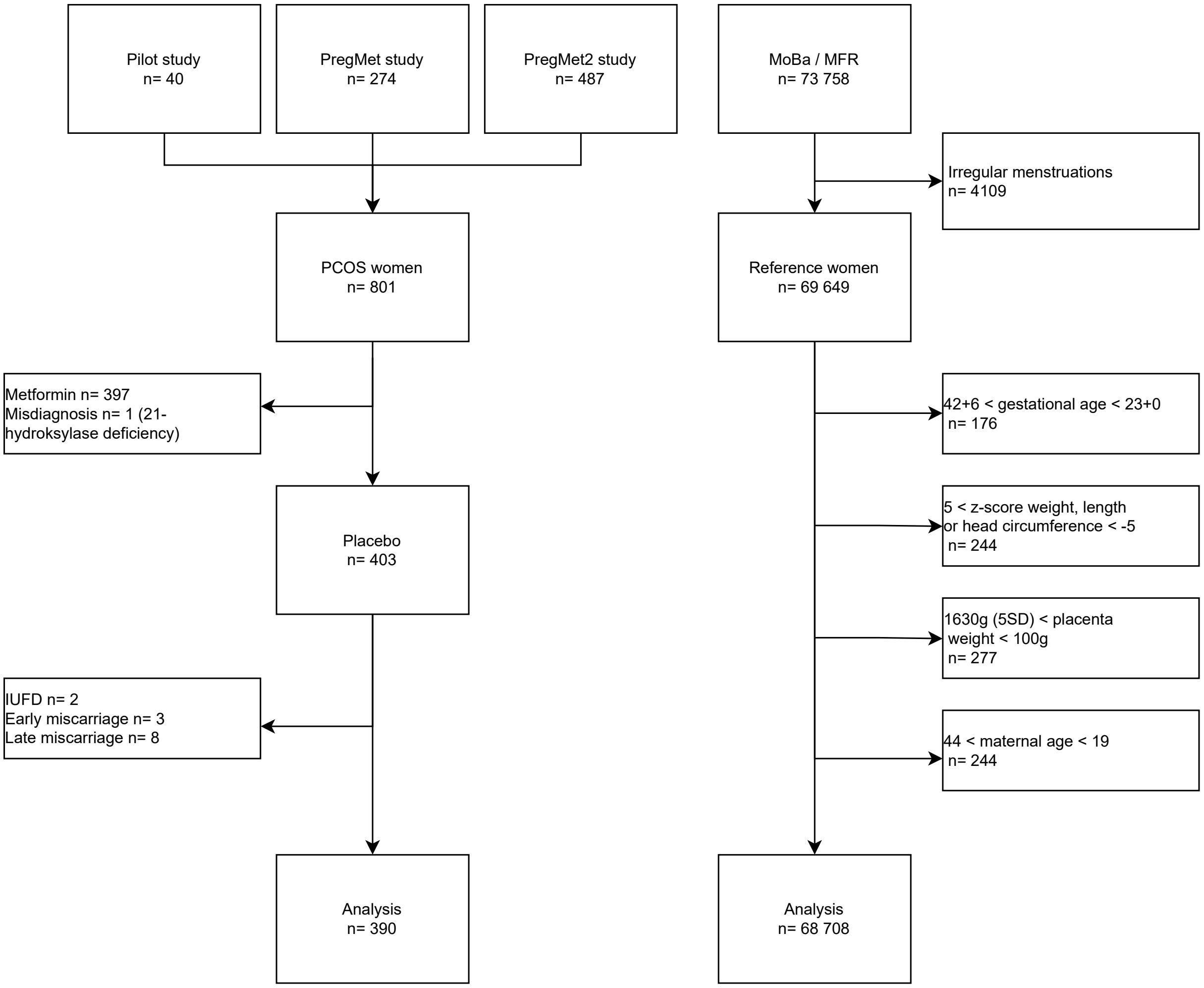

IUFD; intrauterine fetal death; MBRN: the Medical Birth Registry; MoBa: the Norwegian Mother, Father and Child Cohort Study; PCOS: polycystic ovary syndrome; SD: standard deviation.

© 2024 Talmo MSA et al. *JAMA Network Open*.
