# Supplementary material for: Growth Restriction in the Offspring of Mothers With Polycystic Ovary Syndrome
Source: JAMA Netw Open. 2024 Aug 27;7(8):e2430543. doi: 10.1001/jamanetworkopen.2024.30543 (PMC11350484; doi:10.1001/jamanetworkopen.2024.30543)
Supplement: Supplement 2. — Data Sharing Statement [file jamanetwopen-e2430543-s002.pdf]

Data Sharing Statement

Talmo. Growth Restriction in the Offspring of Mothers With Polycystic Ovary Syndrome. *JAMA Netw Open*. Published August 27, 2024. doi:10.1001/jamanetworkopen.2024.30543

Data
Data available: No

Additional Information
Explanation for why data not available: For the reference population: Data from the Norwegian Mother, Father and Child Cohort Study and the Medical Birth Registry of Norway used in this study are managed by the national health register holders in Norway (Norwegian Institute of public health) and can be made available to researchers, provided approval from the Regional Committees for Medical and Health Research Ethics (REC), compliance with the EU General Data Protection Regulation (GDPR) and approval from the data owners. The consent given by the participants does not open for storage of data on an individual level in repositories or journals. Researchers who want access to data sets for replication should apply through helsedata.no. Access to data sets requires approval from The Regional Committee for Medical and Health Research Ethics in Norway and an agreement with MoBa. For the PCOS population: Individual participant data that underlie the results reported in this article, after de-identification, and the study protocol will be available on a collaboration basis for individual participant data meta¬analyses. Proposals should be directed to eszter.vanky@ntnu.no.
